# Supplementary material for: Human theca arises from ovarian stroma and is comprised of three discrete subtypes
Source: Commun Biol. 2023 Jan 4;6:7. doi: 10.1038/s42003-022-04384-8 (PMC9812973; doi:10.1038/s42003-022-04384-8)
Supplement: Supplementary file 2 — Supplementary Information [file 42003_2022_4384_MOESM2_ESM.pdf]

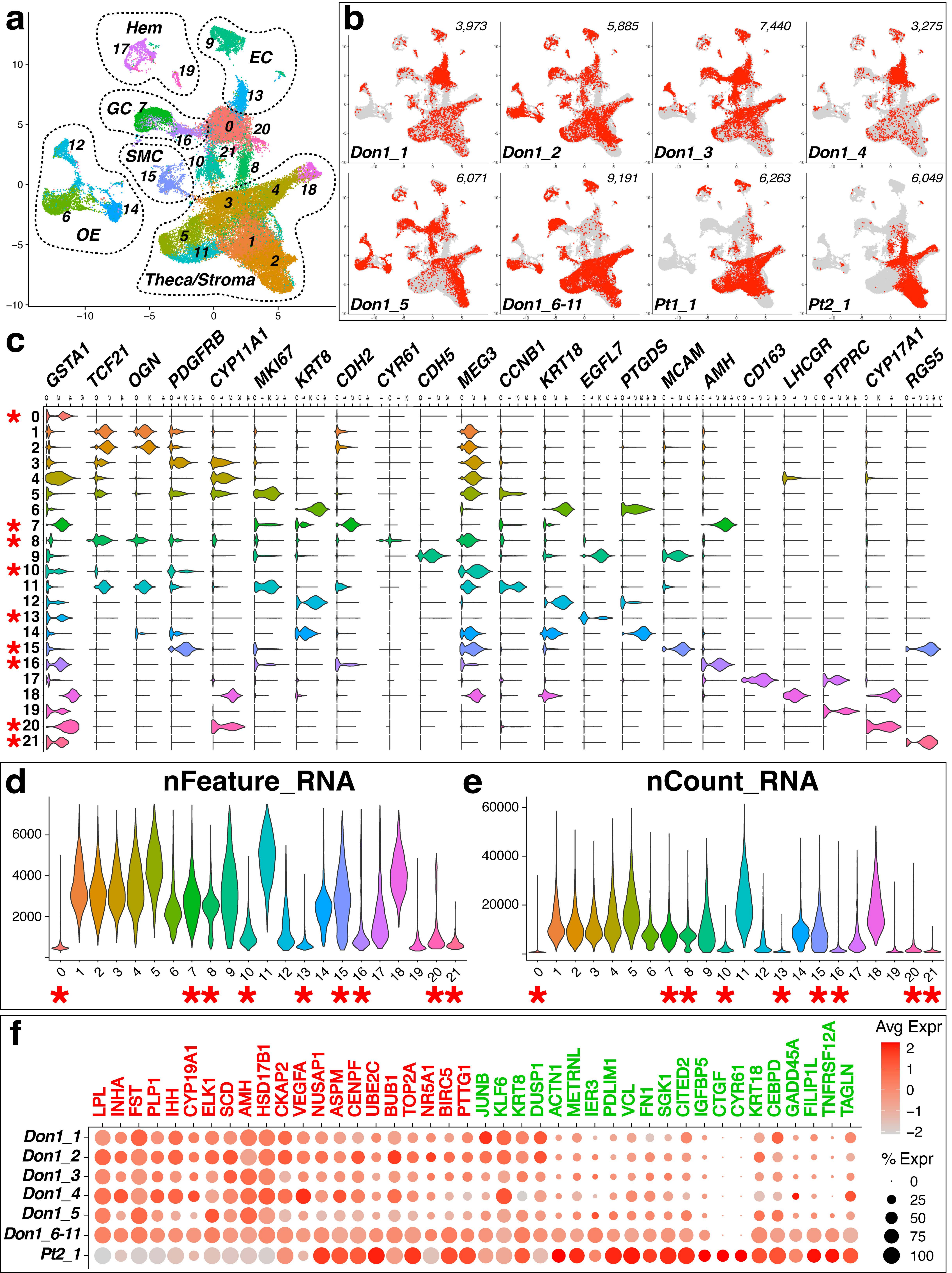
a
Hem
17
19
9
EC
13
GC 7
0
20
16
12
SMC
15
10
21
8
18
4
3
14
6
OE
5
11
1
2
Theca/Stroma
b
3,973
Don1_1
5,885
Don1_2
7,440
Don1_3
3,275
Don1_4
6,071
Don1_5
9,191
Don1_6-11
6,263
Pt1_1
6,049
Pt2_1
c
GSTA1
TCF21
OGN
PDGFRB
CYP11A1
MKI67
KRT8
CDH2
CYR61
CDH5
MEG3
CCNB1
KRT18
EGFL7
PTGDS
MCAM
AMH
CD163
LHCGR
PTPRC
CYP17A1
RGS5
d
nFeature_RNA
e
nCount_RNA
f
LPL
INHA
FST
PLP1
IHH
CYP19A1
ELK1
SCD
AMH
HSD17B1
CKAP2
VEGFA
NUSAP1
ASPM
CENPF
UBE2C
BUB1
TOP2A
NR5A1
BIRC5
PTTG1
JUNB
KLF6
KRT8
DUSP1
ACTN1
METRNL
IER3
PDLIM1
VCL
FN1
SGK1
CITED2
IGFBP5
CTGF
CYR61
KRT18
CEBPD
GADD45A
FILIP1L
TNFRSF12A
TAGLN
Avg Expr
% Expr
Don1_1
Don1_2
Don1_3
Don1_4
Don1_5
Don1_6-11
Pt2_1

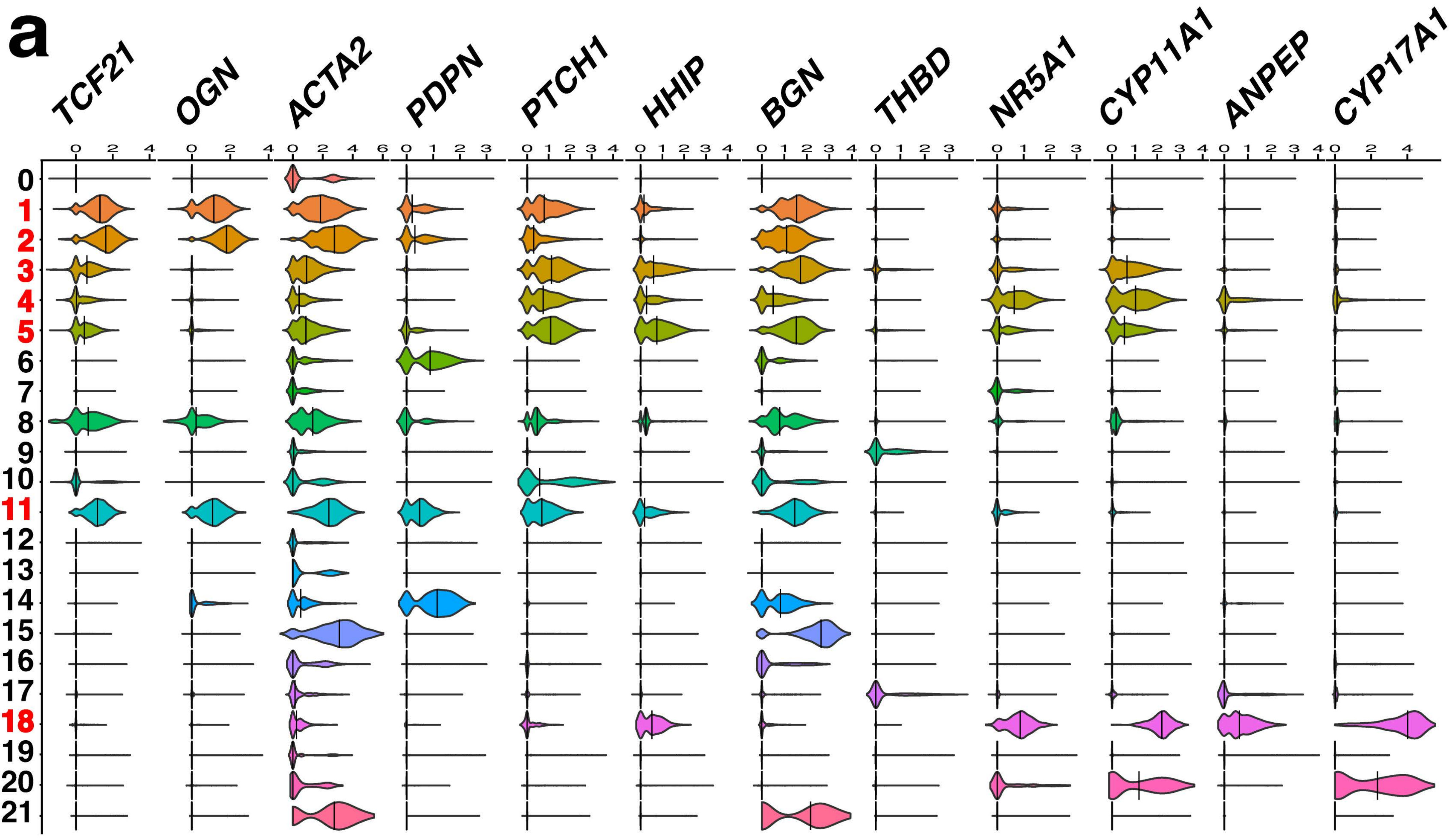
a
TCF21
OGN
ACTA2
PDPN
PTCH1
HHIP
BGN
THBD
NR5A1
CYP11A1
ANPEP
CYP17A1
0
1
2
3
4
5
6
7
8
9
10
11
12
13
14
15
16
17
18
19
20
21

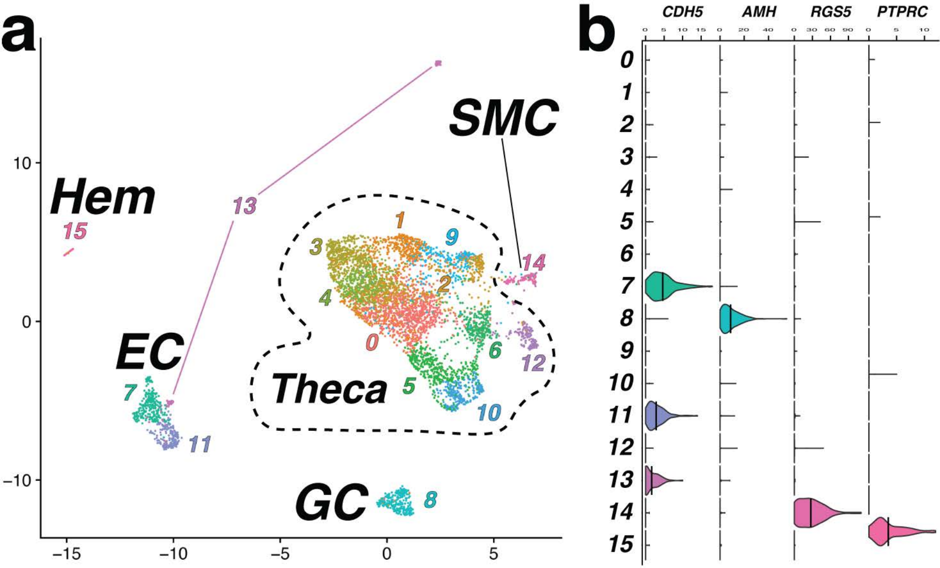
a
b
Hem
15
13
SMC
14
1
9
3
2
4
0
6
12
EC
7
Theca
5
10
11
GC
8
10
0
−10
−15
−10
−5
0
5
CDH5
AMH
RGS5
PTPRC
0 5 10 15
0 20 40
0 30 60 90
0 5 10
0
1
2
3
4
5
6
7
8
9
10
11
12
13
14
15

Supplemental Table 1. Description of ovarian tissues sources and use.

Sample Designation	Tissue Source	Age (years)	Medical History/Cause of death	Material	Usage (Library name)	Figures
Don1	Tissue donor	31	Stroke	Single cell preparations from isolated antral follicles (fresh)	scRNASeq (Don1_1, Don1_2, Don1_3, Don1_4, Don1_5, Don_6-11)	Supp Fig 1a-f, Fig 1a-b, Fig 2a
				Frozen sections of fixed fresh tissue	Immunolabeling	Fig 2b-d, Fig 2g-I, Fig3a-d, Fig 3f, Fig 4a
					RNA in situ hybridization	Fig 1d-g
Pt1	Fertility Preservation	21	Acute lymphocytic leukemia	Single cell preparations from isolated antral follicles (fresh)	scRNASeq (Pt1_1)	Supp Fig 1a-f, Fig 1a-c, Fig 2a
Pt2	Fertility Preservation	24	Chronic myeloid leukemia	Single cell preparations from isolated antral follicles (fresh)	scRNASeq (Pt2_1)	Supp Fig 1a-f, Fig 1a-c, Fig 2a
Pt3	Fertility Preservation	23	Acute myeloid leukemia	Single cell preparations from isolated antral follicles (fresh)	snRNASeq/scATACSeq (Pt3_1)	Fig 5a-f
Don2	Tissue donor	28	Stroke	Frozen sections of xenotransplanted fixed cortical tissue	Xenotransplantation for proliferation studies (n=4 tissue pieces, n=2 mice)	Fig 4b-h
				Frozen sections of fixed fresh tissue	RNA in situ hybridization	Fig 3e, Fig 3g
					Immunolabeling	Fig 2e-f

Supplementary Material

Supplemental Figure 1. Single cell distribution and Quality Control of global scRNASeq analysis. (a) UMAP plot of all cell populations submitted for scRNASeq with grouping of cell clusters into six main follicular populations; SMC = Smooth muscle cell, GC = Granulosa cell, OE = Ovarian epithelium, Hem = Hematopoietic, EC = Endothelial cell. (b) Distribution of cells on the aggregate UMAP plot from each library preparation showing the sample designation and cell number retained for final analysis. (c) Violin plots showing expression of representative markers used to identify main follicular populations by Seurat cluster; clusters with low features and/or counts are designated by red asterisks. (d-e) Violin plots showing number of RNA features (d) and RNA counts (e) per Seurat cluster. (f) Dot plot showing expression levels of genes associated with an atretic GC phenotype within the identified GCs from each library. Genes in red are downregulated in atretic follicles, while genes in green are upregulated in atretic follicles.

Supplemental Figure 2. Expression level of marker panel genes in theca and non-TCs from the ovary. (a) Violin plots of transcripts that were used to define unique theca sub-types (Fig 2a) showing expression levels across all cell clusters isolated from follicles (SuppFig 1a). Clusters that were included in the theca/stroma group are highlighted in red.

Supplemental Figure 3. (a-b) UMAP plot (a) showing predicted Seurat clusters based on snRNASeq and scATACSeq profiles of isolated cells, with violin plots of common gene expression (b) used to identify main populations designated in (a).

Supplemental Table 1. Description of ovarian tissue sources and use.

Supplemental Data 1. List of significantly differentially expressed transcripts by Seurat cluster. Genes differentially expressed by LogFC ≥0.25 globally among Seurat clusters from either the “All cells” comparison (SuppFig 1) or the curated sub-analysis of theca/stroma cells (Fig 1 and 2) are listed.

Supplemental Data 2. Values used for quantification of cell proliferation in EdU/CldU treated, xenograft-bearing mice.
