# Supplementary material for: Human theca arises from ovarian stroma and is comprised of three discrete subtypes
Source: Commun Biol. 2023 Jan 4;6:7. doi: 10.1038/s42003-022-04384-8 (PMC9812973; doi:10.1038/s42003-022-04384-8)
Supplement: Supplementary file 3 — Description of Additional Supplementary Files [file 42003_2022_4384_MOESM3_ESM.pdf]

Description of Additional Supplementary Files

File name: Supplementary Data 1

Description: Source data behind graphs presented in Figures 2 and 5.

File name: Supplementary Data 2

Description: Source data behind graphs in Figure 4d-f and h.
