# Supplementary material for: Human theca arises from ovarian stroma and is comprised of three discrete subtypes
Source: Commun Biol. 2023 Jan 4;6:7. doi: 10.1038/s42003-022-04384-8 (PMC9812973; doi:10.1038/s42003-022-04384-8)
Supplement: Supplementary file 6 — Reporting Summary [file 42003_2022_4384_MOESM6_ESM.pdf]

nature portfolio

Corresponding author(s): *Double-blind peer review submissions: write DBPR and your manuscript number here instead of author names.*

Last updated by author(s): *YYYY-MM-DD*

Reporting Summary

Nature Portfolio wishes to improve the reproducibility of the work that we publish. This form provides structure for consistency and transparency in reporting. For further information on Nature Portfolio policies, see our Editorial Policies and the Editorial Policy Checklist.

Statistics

For all statistical analyses, confirm that the following items are present in the figure legend, table legend, main text, or Methods section.

n/a	Confirmed	
☐	☒	The exact sample size (*n*) for each experimental group/condition, given as a discrete number and unit of measurement
☐	☒	A statement on whether measurements were taken from distinct samples or whether the same sample was measured repeatedly
☐	☒	The statistical test(s) used AND whether they are one- or two-sided<br>*Only common tests should be described solely by name; describe more complex techniques in the Methods section.*
☒	☐	A description of all covariates tested
☒	☐	A description of any assumptions or corrections, such as tests of normality and adjustment for multiple comparisons
☒	☐	A full description of the statistical parameters including central tendency (e.g. means) or other basic estimates (e.g. regression coefficient) AND variation (e.g. standard deviation) or associated estimates of uncertainty (e.g. confidence intervals)
☒	☐	For null hypothesis testing, the test statistic (e.g. *F*, *t*, *r*) with confidence intervals, effect sizes, degrees of freedom and *P* value noted<br>*Give P values as exact values whenever suitable.*
☒	☐	For Bayesian analysis, information on the choice of priors and Markov chain Monte Carlo settings
☒	☐	For hierarchical and complex designs, identification of the appropriate level for tests and full reporting of outcomes
☒	☐	Estimates of effect sizes (e.g. Cohen's *d*, Pearson's *r*), indicating how they were calculated

Our web collection on statistics for biologists contains articles on many of the points above.

Software and code

Policy information about availability of computer code

Data collection	*Provide a description of all commercial, open source and custom code used to collect the data in this study, specifying the version used OR state that no software was used.*
Data analysis	All analysis of single cell RNA sequencing data was performed using the Seurat program in R-Studio

For manuscripts utilizing custom algorithms or software that are central to the research but not yet described in published literature, software must be made available to editors and reviewers. We strongly encourage code deposition in a community repository (e.g. GitHub). See the Nature Portfolio guidelines for submitting code & software for further information.

Data

Policy information about availability of data

All manuscripts must include a data availability statement. This statement should provide the following information, where applicable:

- Accession codes, unique identifiers, or web links for publicly available datasets
- A description of any restrictions on data availability
- For clinical datasets or third party data, please ensure that the statement adheres to our policy

Data Availability
All data needed to evaluate the conclusions in this paper are present in the paper and/or the Supplementary Materials. Single cell multi-omics data is available on

GEO. The data described in this manuscript is listed on GEO under record number GSE192722.

Human research participants

Policy information about studies involving human research participants and Sex and Gender in Research.

Reporting on sex and gender	Human ovarian material was obtained from two sources: Braindead organ donors (Don) or Fertility Preservation Patients (Pt). For organ donors whole bilateral ovaries were obtained from brain-dead organ donors with consent, as a part of a collaboration with the International Institute for the Advancement of Medicine. Tissue from braindead organ donors obtained with informed consent of the donor or family. For Fertility Preservation Patients, we obtained discarded material from patients with informed consent and with approval of the Weill Cornell Institutional Review Board – Study title: Low temperature preservation of ovarian tissue; Study number: 0803009702.
Population characteristics	Women of reproductive age undergoing chemo/radiotherapy for cancer treatment.
Recruitment	All patients presenting to our clinic for ovarian cryopreservation were considered
Ethics oversight	Weill Cornell Institutional Review Board – Study title: Low temperature preservation of ovarian tissue; Study number: 0803009702.

Note that full information on the approval of the study protocol must also be provided in the manuscript.

Field-specific reporting

Please select the one below that is the best fit for your research. If you are not sure, read the appropriate sections before making your selection.

☒ Life sciences ☐ Behavioural & social sciences ☐ Ecological, evolutionary & environmental sciences

For a reference copy of the document with all sections, see nature.com/documents/nr-reporting-summary-flat.pdf

Life sciences study design

All studies must disclose on these points even when the disclosure is negative.

Sample size	Sample analysis was performed in at least triplicate, with post-facto analysis of statistical significance.
Data exclusions	No data exclusions
Replication	Replicates values were analyzed by Wilcoxx Sum rank test and only values that reached a threshold of statistical significance were considered for follow-up analysis
Randomization	N/A
Blinding	Investigators were not blinded to the group allocation for scRNASeq analysis. Quantification of EdU,CldU, Ki67 values were performed by inestigators that were blinded to the condition they were assessing.

Reporting for specific materials, systems and methods

We require information from authors about some types of materials, experimental systems and methods used in many studies. Here, indicate whether each material, system or method listed is relevant to your study. If you are not sure if a list item applies to your research, read the appropriate section before selecting a response.

Materials & experimental systems

n/a	Involved in the study	
☐	☒	Antibodies
☐	☒	Eukaryotic cell lines
☒	☐	Palaeontology and archaeology
☐	☒	Animals and other organisms
☒	☐	Clinical data
☒	☐	Dual use research of concern

Methods

n/a	Involved in the study	
☒	☐	ChIP-seq
☐	☒	Flow cytometry
☒	☐	MRI-based neuroimaging

Antibodies

Antibodies used

Antibody Name Company Catalogue #.
Alexa Fluor 488 anti-human CD45 (clone HI30) BioLegend
Cat#304019

DAPI Invitrogen
Cat#D1306

PE anti-human (CD13) ANPEP (clone WM15) BioLegend
Cat#301703

APC anti-human (CD13) ANPEP (clone WM15) eBiosciences Cat# 17-0138-41
PE anti-human CD31 (clone WM59)
BioLegend
Cat#303106;

FITC anti-human CD55 (clone JS11) BioLegend
Cat#311306; RRID:AB_314863
FITC anti-human CD99 (clone 3B2/TA8)
BioLegend
Cat#371303
APC anti-human CD99 (clone 3B2/TA8)
BioLegend
Cat#371308
TCF21 Polyclonal Antibody Invitrogen Cat# PA5-53031
Human/Mouse/Rat alpha- Smooth Muscle Actin Antibody R&D Systems Cat# MAB1420

Alexa Fluor 647 anti-human CD141(Thrombomodulin) – Clone (M80) BioLegend Cat# 344123
Gli1 Polyclonal Antibody Invitrogen Cat# PA5-72967
Human/Mouse Patched 1/PTCH (First Extracellular Loop) Antibody R&D Systems Cat# MAB41051

CYP11A1 (D8F4F) Rabbit mAb Cell Signaling Technologies Cat# 14217S
Human Endoglin/CD105 Alexa Fluor 488-conjugated Antibody R&D Systems Cat# FAB10971G
CYP17A1 (E6A7G) XP Rabbit mAb Cell Signaling Technologies Cat# 94004S
PE anti-human Podoplanin (PDPN) Antibody BioLegend Cat# 337003
Human CD34 APC-conjugated Antibody R&D Systems Cat# FAB7227A

Anti-BrdU antibody [BUI/ 75 (ICR1)] Abcam Cat# ab6326
Pe anti-human CD141(Thrombomodulin) – Clone (M80) BioLegend Cat# 344103
Anti-Ki67 antibody Abcam Cat #ab15580
Anti-CYP17 Antibody (D-12) Santa Cruz Cat# sc-374244
Alexa Fluor Plus 647 goat anti-rabbit Invitrogen Cat# A32733

Alexa Fluor 647 goat anti-rat
Invitrogen Cat# A21247

Alexa Fluor 488 donkey anti-rabbit IgG
Life Technologies Cat# A21206

Alexa Fluor 488 goat anti-rat
Invitrogen Cat# A11006

Alexa Fluor 555 donkey anti-rabbit IgG
Life Technologies Cat# A31572

Anti-hBiglycan affinity purified Goat IgG R&D Systems Cat# AF 2667
PE anti-CD105 (Endoglin) Biolegend Cat# 800503
Alexa Fluor 488 donkey anti-goat IgG Life technologies Cat# A11055

Validation

Validation of each antibody is described in the product information and is available from the manufacturer for each antibody listed above.

Eukaryotic cell lines

Policy information about cell lines and Sex and Gender in Research

Cell line source(s)

Endothelial cells
Human ECs were obtained from Angiocrine Bioscience and were originally isolated from neonatal Umbilical Vein (HUVEC) as described62 under an IRB approved protocol for use of discard biological material. HUVEC were isolated and expanded for three passages in endothelial cell growth medium before cryopreservation.

Authentication	Cells are authenticated by qualification of endothelial-specific surface markers: VE-cadherin, CD31 and CD34.
Mycoplasma contamination	Cells are tested for mycoplasma contamination.
Commonly misidentified lines (See ICLAC register)	N/A

Animals and other research organisms

Policy information about studies involving animals; ARRIVE guidelines recommended for reporting animal research, and Sex and Gender in Research

Laboratory animals	Xenografts were performed in female immune-compromised NOD scid gamma (NSG) mice.
Wild animals	N/A
Reporting on sex	All xenografts were performed in female mice, as increased androgens in males could adversely influence follicular development. Additionally, previous studies of human ovarian xenografts suggest that oophorectomy of host mice, and resultant induction of menopausal levels of gonadotropins, can potentially have a beneficial effect on xenograft productivity.
Field-collected samples	N/A
Ethics oversight	All procedures were approved, and experiments were performed in accordance with the guidelines and regulations of the Institutional Animal Care and Use Committee (IACUC) of Weill Cornell Medicine (IACUC Protocol #2014-0008 – Assessment of angiogenic and hematopoietic tissue in mouse).

Note that full information on the approval of the study protocol must also be provided in the manuscript.

Flow Cytometry

Plots

Confirm that:

☒ The axis labels state the marker and fluorochrome used (e.g. CD4-FITC).

☒ The axis scales are clearly visible. Include numbers along axes only for bottom left plot of group (a 'group' is an analysis of identical markers).

☒ All plots are contour plots with outliers or pseudocolor plots.

☒ A numerical value for number of cells or percentage (with statistics) is provided.

Methodology

Sample preparation	Cell pellets were washed with DPBS (Gibco) and resuspended in blocking solution containing either antibodies that were directly conjugated to CD99 and CD45 for the cells obtained from initial Accutase dissociation, or CD55 and ANPEP for the cell pellet obtained following Collagenase/Dispase dissociation. After incubation for 10 minutes at 4°C, cells were washed, centrifuged, and resuspended in FACS buffer containing DAPI. Cells were run on a FACSJazz (BD) with collection and validation of the obtained fractions following initial sort. Purified cell population were washed, centrifuged, and submitted for either scRNASeq or combined snRNASeq/scATACSeq. In addition, for the investigation of the THBD population a fraction of the cell pellet obtained from the secondary Collagenase/Dispase dissociation was stained for CD34, THBD and ANPEP. Following cell sorting fresh single cell suspensions containing GCs (CD99+CD45neg) or TCs (CD55+ANPEP+/neg) were submitted for single-cell RNA sequencing(scRNASeq) or Combined Single-nucleolar RNA sequencing(snRNASeq) and Single-cell Assay for transposase-accessible Chromatin (ATACSeq) with high-throughput sequencing.
Instrument	BD FACS Jazz
Software	FACS Diva
Cell population abundance	Samples were confirmed post sort to be >98% pure
Gating strategy	Following cell sorting fresh single cell suspensions containing GCs (CD99+CD45neg) or TCs (CD55+ANPEP+/neg) were submitted for single-cell RNA sequencing(scRNASeq) or Combined Single-nucleolar RNA sequencing(snRNASeq) and Single-cell Assay for transposase-accessible Chromatin (ATACSeq) with high-throughput sequencing.

☒ Tick this box to confirm that a figure exemplifying the gating strategy is provided in the Supplementary Information.
